# Baby Clinic Experiences

**Published:** 1920-06-19

**Authors:** 


### Baby Clinic Experiences.

SOME useful points were made in a paper read at a sessional meeting of the Royal Sanitary Institute in Lincoln on the subject of the development of maternity and child welfare work in a smaller county borough. Dr. C. J. Coleman, the Medical Officer of Health at Lincoln, who was the author of the paper, reviewed the outstanding facts of infant mortality—among legitimates and illegitimates—dealt briefly with some of the various causes and influences, and contended that it is an imperative duty to preserve infant national life; in fact, it is one of the chief national-duties. In Lincoln (whose organisation was described by Dr. Coleman) the penny rate produces about £1,000, and is not sufficient for very pretentious enterprise. Infant welfare was first seriously undertaken by a now defunct voluntary society, and in 1910 its duties were undertaken by the corporation. In 1911 a start with an infant clinic was made, and 155 attendances of mothers and babies took place during the first year. In 1915 arrangements were completed for giving medical advice at these clinics, and during that year there were no fewer than 8,849 attendances of mothers.

An amusing account of the vicissitudes of the babies' clinic was given by Dr. Coleman. After one removal from its original starting place it was centred at the Mayor's parlour, but the voices of the babies so disturbed the deliberations in the adjoining committee-room that the babies had to be wheeled off elsewhere. But there they interfered with the medical school work, as they were near the school clinic that had meanwhile been opened, so off they perambulated again, only to be passed along once more on account of military requirements. The military chased them out a second time, but the clinic insisted on thriving despite these adversities, and after the first Lincoln baby week in 1917 a scheme was successfully launched which resulted in the Corporation purchasing a permanent centre for £1,050. The class of mother attending this clinic seems to be above the average, and Dr. Coleman says that on many afternoons from £500 to £700 worth of perambulators may be seen stacked under the verandas.

Dr. Coleman has found from the experience of the clinic that some mothers are only too willing to give as an excuse for weaning their babies at the end of a month or two months that their doctor told them their milk was not suitable. Though he considers these assertions are not always correct, he expresses his opinion that frequently the medical profession has been to blame in not encouraging breast-feeding. He says he himself has the greatest difficulty in making mothers understand that even if they are not enjoying robust health, their own breast milk must be far superior than any artificial substitute. It is a fact that it is often necessary for mothers to persevere when breast-feeding does not seem to be going along smoothly, but too many of them give up before they have given a proper trial; though the wise mother faces these things.

The 1917 baby week having set going the baby clinic, the 1918 baby week focussed attention on the need of a maternity home, which, with the aid of private generosity, was purchased and opened in September last year. There have since been 121 applicants, and the home proves a great boon to the city. Fees extend to two guineas or more a week, and there are also free admissions. The Lincoln activities include the infant welfare centre; a school for mothers with lectures by medical officers and health visitors, and demonstrations in cookery and needlework; district nursing; ante-natal clinic; maternity home; district midwifery; and home helps.

